# Shared Housing Arrangements in Germany—An Equitable Alternative to Long Term Care Services beyond Homes and Institutions?

**DOI:** 10.3390/ijerph15020342

**Published:** 2018-02-14

**Authors:** Lorraine Frisina Doetter, Achim Schmid

**Affiliations:** Global Dynamics of Social Policy, CRC 1342, Project A04 & The Research Center on Inequality and Social Policy, Department of Health, Long Term Care, and Pensions at the University of Bremen, 28359 Bremen, Germany; achim.schmid@uni-bremen.de

**Keywords:** long term care, shared housing arrangements, Germany, equity

## Abstract

Given the saliency of socio-demographic pressures, the highly restrictive definition of “need for care” characterizing the German long-term care system at its foundations in 1994 has since been subject to various expansionary reforms. This has translated into greater interest in innovative care models that provide more choice and flexibility to beneficiaries. One such model is ‘shared housing arrangements’ (“ambulant betreute Wohngemeinschaften”), where a small group of people rent private rooms, while sharing a common space, domestic support, and nursing care. Using interview and secondary data, this study examines the potential for such arrangements to provide an equitable alternative to care that is accessible to a larger population of beneficiaries than presently seen in Germany.

## 1. Introduction

The German long-term care (LTC) system was introduced in 1994, as a result of strong economic and demographic pressures including the rise in an ageing population, low birthrates since 1965, and the feminization of the workforce which rendered the female care giver model increasingly obsolete [1]. With it, the youngest branch of social security was established within the German welfare state. Following the model of social insurance first created under Bismarck during the late 19th century, the German LTC system relies on compulsory salary-based contributions shared equally by employers and employees and provides beneficiaries with a legal entitlement to services irrespective of their socio-economic background. Instead, benefits correspond with the beneficiary’s “need for care”, an entitlement criterion initially narrowly defined as a means of strict cost control. Amongst the system’s various objectives, providing social security against the risk of needing care in a manner that is akin to insurance against illness, accidents, and unemployment, and protecting income in old age, is central (see Table 1). In recent years, the issue of improving access to services through a new definition of entitlement and as a matter of social justice has also been a key policy goal, particularly to address the care needs of those living with dementia [2]. Dementia, regarded as one of the most costly brain disorders [3] and typically leading to institutionalization [4], is expected to affect three million people in Germany and 115 million people worldwide in the year 2050 [5]. Germany’s response to this mounting demographic challenge has been to redefine care level grades through the use of a new assessment instrument that better accounts for cognitive impairment [6,7]. This has been accompanied by dramatic increases in cash and in-kind benefits across nearly the entire board, as well as growing interest and investment in innovative models of care that can provide more choice and flexibility to beneficiaries, while reducing the need for costly institutionalization.

One model that has gained special attention since first emerging in 1995 in Germany in the city of Berlin [9] is shared housing arrangements (“ambulant betreute Wohngemeinschaften” or SHAs), in which a limited number of six to eight people in need of care rent private rooms in ordinary apartment buildings, while sharing a common space, domestic support, and access to nursing care. Argued as particularly well-suited to the needs of dementia sufferers who require a more familiar environment than found in traditional nursing homes [10], the concept aims to provide a small-scale, home-like facility with ample leeway for individual activities and autonomy [9]. SHAs seek to engage residents in meaningful daily routines and activities such as cooking and cleaning. Alongside the purchase of professional care, they also rely on the social involvement and support of relatives, friends, and community volunteers [11]. SHAs are premised especially on the notion that family involvement is essential to the well-being of care dependents, an assumption supported by evidence comparing the quality of life of residents within SHAs that have different frequencies in the number of visits by relatives [11].

Beyond helping to combat social isolation in old age, family member involvement is said to allow for important biographical information about the care dependent to be passed on to service professionals, thereby allowing for greater person-centered care to be delivered [10,11,12]. Family inclusion also corresponds with the wishes of relatives who generally want to stay involved in the care giving process despite living apart from the afflicted [11,13]. Research suggests that the concept of small-scale living arrangements provides family members with a lessened burden of care but with greater satisfaction compared to that of traditional nursing facilities [11,13,14]. It bears noting, however, that the integration of family members into the SHA care scheme has, in practice, proven to be challenging due to, amongst other factors, the absence of close relatives that are still alive or living in the area [11]. Given the emphasis placed on family involvement, the SHA concept may, therefore, create a de-facto barrier to access for care dependents that are single or childless.

In view of the postulated advantages associated with this care model, particularly from the standpoint of family members, as well as the introduction of financial incentives by the state for their establishment [15], SHAs have grown in number over the past 15 years: whereas the report of the Kuratorium Deutsche Altershilfe estimated 143 residences in 2003 [16], 200 residences were estimated in 2006 [17], and 3121 residences are estimated to presently operating across Germany [18]. Still, compared to more conventional care settings (i.e., at-home and institutional care), the number of SHAs remains modest [7]. Moreover, a large concentration of SHAs are located in Berlin (594 residences in year 2015) [18], suggesting that the concept may not travel well beyond the context in which it first emerged in Germany [11]. This geographic bias is also mirrored in the data available on the quality of care and quality of life of residents within such arrangements: most evaluation studies refer to residential groups for LTC-patients with dementia in Berlin [19]. These studies do not evidence a significant difference amongst residents across care settings (SHAs versus nursing home facilities) [19]. Meanwhile, biases also emerge in terms of the type of population of residents: although any beneficiary of LTC services is entitled to receive care within an SHA setting, most residents tend to be female, around the age of 80 years, and suffering with dementia [11]. Economically speaking, residents tend to be of a middle class background, although social assistance can be used to support access to SHAs for low income care dependents [15]. Access, however, can be complicated by the policies in place by state municipalities responsible for social assistance payments that may apply various standards and restrictions for assessing financial need associated with SHA residence [15]. This point will be addressed in greater detail in a subsequent section of the study. Taken together, many unanswered questions as to the applicability of SHAs to a wider scope of beneficiaries (e.g., males; varying ages and morbidities; the childless; low socio-economic background etc.) and areas (i.e., rural and urban) emerge, which fundamentally concern the equity of this care model.

In an effort to identify the potential institutional barriers, conceptual limitations, and implementation hurdles surrounding SHAs, the present study begins by analyzing the legal provisions in place to support the set up and access to services within this care setting. Relying on data from expert interviews with three SHA-providers and one LTC nurse involved in managing SHA-care, we then examine how this model has been implemented in three regions of the country—namely, Berlin and other parts of East Germany, the city-state of Bremen in the Northwest, and the state or Land of Baden-Württemberg in the Southwest. We are especially interested in distinguishing the role of specific factors and challenges that either aid or hinder beneficiaries’ access to SHAs, while also exploring the more general impediments to implementation emerging so far. In doing so, the study aims to provide evidence in response to the question, are SHAs an equitable alternative to LTC services beyond homes and institutions?

## 2. Materials and Methods

The present study is exploratory in kind, providing a novel analysis on the equity of SHAs as a model for LTC in Germany. By equity, we refer to both the applicability and accessibility of this concept to a broader spectrum of care dependents, irrespective of their socio-economic background, morbidity, family status (married or single, with or without children), or geographic location (rural versus urban). In addressing the equitability of this care model, we also ask whether SHAs are a viable option beyond a small group and the context of beneficiaries.

The research that follows is qualitative and organized along two steps: first, a legal analysis is conducted relying on primary documents that specify the incentives and regulatory conditions in place by the state for the establishment and operation of SHAs across Germany. This provides us with necessary insight into the institutional landscape defining the so-called ‘rules of the game’ in place to support, but also constrain the development and, consequently, access to SHAs. Second, an analysis of expert opinion is carried out through the use of semi-structured interviews with three providers of SHA residences across three different regional contexts, as well as with one LTC nurse, in order to identify challenges and opportunities involved in the establishment and operation of SHAs, as these may come to bear on the de-facto access of this care concept. We also ask experts to reflect upon the potential of expanding the SHA model to become a more mainstream choice for providing LTC services in the future.

More specifically, the present study includes data obtained from four semi-structured interviews carried out in person (two interviews) and by telephone (two interviews) with informed participants and in line with federal data protection laws regarding consent, data gathering, processing, and storage. In addition, clearance was obtained from the Ethics Commission of the University of Bremen (issued in writing on 12 Feb. 2018 for the present study “Shared Housing Arrangements in Germany”). The duration of interviews ranged from 45 to 90 min, depending on the length of the respondent’s answers to a predefined set of questions (to be discussed). Interviews were recorded, transcribed verbatim in a Word document for analysis, and subsequently anonymized for reporting purposes here. The experts included in the study refer to the following roles and organizations:

- Provider A is a charitable foundation with about 1400 employees based in Baden-Württemberg, Germany. The foundation offers places in nursing homes, as well as outpatient care, day care, assisted living, and SHAs on an inpatient and outpatient basis.
- Provider B is a home care service company located in Berlin and East Germany with about 1800 employees providing assisted living, SHAs, and in-home nursing care.
- Provider C is a charitable organization in Bremen in north-west Germany, with about 2000 employees focusing on home care services: assisted living, SHAs, and at-home nursing care.
- Provider D is a homecare service company responsible for several residential groups in Berlin.

While providers A, B, and C are represented by managers, D is represented by a qualified nurse. Given the limited number of interviewees included in the study, representativeness of the entire population of SHA providers and care professionals is not possible. However, by focusing on a small sample, in-depth interviews could be carried out with experts representing large provider-organizations across various geographic areas within Germany. This helps serve the exploratory purpose of the study.

Regarding the content of the semi-structured interviews, experts were asked to respond to open-ended questions, thereby providing them with ample space to reflect and generate their own input on anything they found relevant to the topic. Interviews focused on the following questions (translated from German):

- Which groups of care-dependents tend to be (over)represented in (a) SHAs and (b) other forms of assisted living compared to more traditional models of care such as services delivered within (c) the beneficiary’s home or in (d) a nursing care facility?
- Can differences be attributed to any of the following characteristics of the beneficiary?
  ○ Economic background
  ○ Social position (educational background or social class)
  ○ Family background (marital status, children, other family relations)
  ○ Ethnic background
  ○ Care level grade or level of dependency and type of illness or condition (somatic versus cognitive)
  ○ Age and sex
  ○ Geographic location (urban versus rural)
- At present, the number of care-dependents living in SHAs across Germany is estimated to be 25,000 to 30,000 people. Where do you see the potential and limits for the expansion of this concept to reach a larger pool of beneficiaries?
- Which ambulatory care models for group living (i.e., SHAs, assisted living, other forms of outpatient care such as daycare centers, etc.) do you believe have the greatest potential for growth in Germany?

Once interview data was collected, responses were carefully read through for meaning and thematically analyzed (without recourse to formal coding) with a view to the study’s definition of equity. Given the empirical focus on providers (total of three out of four experts), we acknowledge the potential for a bias to emerge in an overestimation of the viability of the SHA model that may reflect a business interest on the part of the interviewees. However, we argue that the provider perspective is nevertheless vital to understanding how this model operates at present. In the interpretation of findings, provider reflections on the future potential of SHAs are weighed against insights offered by a nursing care professional, as well as results of the legal analysis in order to establish a more balanced picture of the care model’s equity and viability.

## 3. Results

### 3.1. Legal Framework—Summary and Analysis 

#### 3.1.1. General Benefits and Provisions under LTC Insurance

A guiding principle of the German LTC insurance is to favor home care and outpatient care services over institutionalized care. This reflects the general preference for independent living in old age. The LTC insurance funds home care through cash allowances (Pflegegeld) for informal care giving (especially by family members) or, alternatively, in-kind benefits provided by professional nursing services (Pflegesachleistungen). Cash and in-kind benefits can also be combined with claims set off against each other. Whereas in the case of home care, out-of-pocket payments do not generally have a role in financing, for nursing home care, room and board are not covered by LTC insurance and care costs are covered up to a ceiling (the personal share is calculated as a unitary value per nursing home irrespective of the individual need for care). For home care, the health insurance funds may also cover additional medical nursing services prescribed by doctors. In nursing homes, the LTC insurance covers in-kind benefits that amount to levels slightly higher than those for outpatient care and graded similarly according to the assessed need for care. For severe cases (e.g., end-of-life care), there is extra-funding of about 150 € per month, while medical nursing care is only financed by the health insurance as an exception during short periods of acute care needs.

#### 3.1.2. Federal Incentives for SHAs

LTC reforms of the last ten years have successively improved the benefits for home care and at the same time promoted SHAs as an alternative care model in Germany. Beginning with the LTC Further Development Act of 2008, increases in the entitlements for outpatient nursing care were introduced. The act also stipulated that the use of day and night care can only be partially offset by a reduction of outpatient care benefits. This translated to benefit-increases of up to 50 percent for nursing concepts combining outpatient care with day and night care. More recently, the Long-Term Care Reorientation Act of 2012 established explicit financial support for residential groups with a monthly supplement of 200 € per resident, allowing for the coordination of care and assistance involved in SHAs. The First Act to Strengthen Long-Term Care taking effect in 2015 further improved benefits for day and night care, which can now be fully claimed in combination with nursing care benefits without ensuing any reduction of either service. The act has also made it easier to claim short-term, holiday, or respite care benefits and has raised the supplement for residential groups. Besides this, the act has introduced grants for the establishment of residential groups and barrier-free homes. Finally, the Second Act to Strengthen Long-Term Care taking effect in January 2017 has increased the level of outpatient nursing care benefits again. It also constrained, however, the combined claim of day and night care benefits with the supplement for residential groups. Nevertheless, the reforms of the last ten years have substantially promoted SHAs (and other concepts of assisted living) by allowing for the accumulation of various entitlements to outpatient services, which has amounted to considerably higher insurance benefits than those available for nursing home care.

#### 3.1.3. State-Level (Länder-Level) Regulation of SHAs

While LTC benefits are regulated and universally defined at the federal level, administrative law governing nursing homes and, to various extents, sheltered housing, assisted living, and SHAs, have been devolved to the German states or Länder. Generally, home care is not affected by these regulations. In order to guarantee standards in outpatient care, however, professional nursing services are supervised by competent bodies belonging to the social and private LTC insurances. By contrast, nursing homes have to comply with far more stringent regulations concerning structural conditions and staff ratios, as well as undergo regular inspections. Usually, SHAs are classified under ‘home care’ and thus remain largely unregulated. In particular, fully autonomous residential groups that are organized independently of professional nursing services and which define and purchase care individually or through joint committees are mostly free of additional state regulation. However, the more that SHAs imply any structural dependence of residents upon providers, the more that state-level administrative law becomes applicable so as to protect the interests of residents in need of care [15]. This is generally the case whenever the providers of housing and assistance also offer nursing services or else constrain the choice and/or volume of nursing services purchased [15]. It is also the case whenever providers determine the daily routines similarly to nursing home care. Moreover, states determine the upward limit of residents allowed in SHAs, which in most cases, amounts to a maximum of 12 persons, conforming with the federal definition of SHAs in the entitlement to the SHA-supplement. Currently, in Hamburg, the limit is set to 10 persons, whereas in six states (Bremen, Brandenburg, Hesse, Saarland, Saxony, and Schleswig-Holstein), a boundary has not been defined.

Where state-regulation (and differences therein) tends to emerge is in the way elements of structural dependence are defined, as well as the nature of regulations applied to SHAs when they are no longer considered ‘home care’ and beyond the scope of administrative law. This can happen when SHAs become too large in terms of the number of residents, or when the boundaries between an SHA and a nursing facility become blurred (e.g., when the two are located in one building or when a nursing service has an office situated within an SHA). There is also some regional variation with respect to regulations concerning the availability of round-the-clock assistance, construction-related housing requirements, and routine controls by the inspectorate, as well as the establishment of a board of representatives, though such regulations are typically less demanding than those for institutionalized care. Hence, SHAs are to some extent free from the regulations applied to nursing homes, which translates to fewer expenses for infrastructure and costs related to conditions set in place by supervisory bodies. As a consequence of being independent of most state administrative law, SHAs are allowed greater flexibility in the allocation of personnel, thereby making it possible to use qualified nursing and other staff more efficiently.

Taken together, the institutional landscape can vary for SHAs across Germany, with various degrees of incentives and constraints in place vis-à-vis the specifics of local administrative law, as well the states’ placement of SHAs along the spectrum of more traditional forms of care (i.e., whether closer to the individual’s home or the institutionalized setting of a nursing facility). States can therefore create an additional layer of incentives or impediments for access to the SHA care model for providers and, by extension, beneficiaries. Concerning the states included in the present study, a range of regulatory environments is represented: Berlin provides a context in which state regulation is more conducive to the creation of autonomous SHAs, with fewer legal restrictions and more generous financial support in place by the social assistance authorities (Sozialhilfeträger) [15]. At the same time, Berlin does not permit provider-organized SHAs to operate in the area—although, this can be circumvented by providers wielding informal control over SHAs. This emerges, for example, when housing and nursing providers tacitly collaborate when SHAS are set up, despite being separately contracted. In the case of Baden Württemberg (BW), state law creates a more restrictive regulatory environment which, on the one hand, permits provider-organized SHAs to operate, but, on the other hand, restricts them to offering nursing services for the SHA; requires them to provide round-the-clock assistance for residents; and subjects them to construction requirements (e.g., room size, bathroom accessibility) and regular supervision. Moreover, resistance on the part of the state’s social assistance authorities to provide financial support to cover costs related to SHAs creates a de facto barrier to access for low socio-economic groups. Finally, Bremen occupies something of a middle position in terms of regulatory environments, compared to Berlin and BW. As in the case of BW, Bremen allows provider-run SHAs to operate, but does not restrict the providers to offer nursing services for the SHA, given that separate contracts for housing and nursing care exist. Contrary to BW, Bremen takes a more supportive role in the creation of SHAs, since the regional nursing home law explicitly allows providers to transform nursing care facilities into small-scale group living units. While residents must have the freedom to choose their preferred nursing care service, the provider of housing and assistance is responsible for the organization and coordination of services and therefore may have some influence on forms of living and care arrangements.

### 3.2. Additional Financial Incentives Affecting SHAs

The LTC benefits system differs between institutionalized care and home care (see Section 3.1.1). Home care, including SHAs, can be organized in such a way that accumulates or combines various types of in-kind benefits: e.g., LTC professional nursing services; medical nursing care; day care; and short-term and respite care. From a provider perspective, this establishes economic incentives to expand SHAs and combinations of assisted living with day care arrangements—an option not available to nursing home facilities, rendering them less attractive to providers. Economic incentives are also related to regional billing rules defined by welfare authorities and LTC insurance funds: e.g., flat charges for coordinating care or collective services within the SHA environment facilitate billing for providers [15]. People in need of care may also profit from such care concepts, as the accumulation of insurance benefits can reduce the personal share of care costs, especially compared to more expensive out-of-pocket financing for institutionalized care. At the same time, where state administrative law requires 24 h assistance to be provided in SHAs, out-of-pocket spending can become costly. By way of example, an evaluation study conducted in North Rhine-Westphalia (NRW) in 2015 revealed monthly costs of about 1300 € for this service [20]. Alongside payments for room and board, expenses related to 24 h assistance can be an impediment for the less well-off to access SHAs. At present, access by recipients of social assistance depends upon regional and even local implementation rules: standardized means testing procedures and billing rules facilitate access in some states, while case-by-case decisions and reluctance to finance extra costs involved with SHAs considerably hinder access in others [15]. Once again, state-level administrative law may condition the impact of federal incentives on the development and availability of SHAs in Germany. Hence, while federally defined legal provisions do not appear to contribute to biases in access, state and local law may create a significant roadblock for differences to emerge based on the socio-economic status of beneficiaries.

### 3.3. Expert Interviews—Summary and Analysis

All interview partners highlighted that access to SHAs differs from access to nursing homes: legally, one must distinguish between fully autonomous SHAs and those organized by care providers. The former establishes a committee consisting of residents and/or their advocates. This committee is responsible for common decisions including the admission of new residents and the choice of providers. Hence, access to the SHA requires the consent of the present residents. Provider-organized SHAs generally also involve a residents’ committee that meets periodically to represent the interests of the beneficiaries, with providers engaging in an advisory capacity. Formally, these committees participate in any decision concerning the residential group. However, in practice, in most SHAs, providers are the main decision-makers and act as gatekeepers shaping the composition of the residential group (Provider D). Criteria for access are first of all dependent upon the design of the SHA: e.g., residential groups for dementia patients; for specific groups of ethnic minorities; for young people in need of care; and for residents of the neighborhood, etc. (all providers). Providers also considered the role that economic background plays amongst residents of SHAs (discussed below). Provider D stated that the gatekeeper role of providers also means that personal contacts between providers and residents or their (legal) advocates often determine the chances to access SHA.

Concerning the economic status of people in need of care, Provider A emphasized the general tendency of the less well-off to choose cash over in-kind benefits in order to increase their pension, as long as they can sustain home care with informal support (Provider A). This same population has also been found to forgo other services such as respite and short-term care, as well as medical nursing care to which they are entitled (Provider A). This may indicate a problem of take-up of care rather than access, per se. Meanwhile Provider B stated that SHAs are no longer restricted to the academic middle class environment in which they developed. Based on all interviews, the role of economic background in determining access to residential groups varies regionally. Regional cost structures, the policies put in place by local welfare authorities, and the local supply and demand for SHAs are important criteria. Provider A located in Baden-Württemberg emphasized the costly requirements for SHAs, mainly 24 h assistance, as an impediment. Access for recipients of social assistance in such regions is unlikely since welfare authorities tend to make case-by-case decisions and often refuse to pay the costs associated with SHAs (e.g., more expensive rent). Therefore, providers have filed several lawsuits to enforce payments by local authorities (Provider A).

Provider A, translated: *With an SHA in Stuttgart, active since 2008, we have filed the third, fourth or fifth law suits. The welfare authority refuses to pay the allowance for assistance calculated according to the high demands of the regional regulations (Gesetz für unterstützende Wohnformen, Teilhabe und Pflege, WTPG) while the German City Council fails to adjust the guidelines for social assistance (…) The welfare authorities are not willing to pay the costs for sustainable and lawful employment conditions.*

Provider D also confirmed the difficulties for the economically weak to access SHAs in a market where demand outnumbers supply. Housing and boarding costs which are not fully covered by social assistance were named as the main impediments (Provider D). Access by social assistance recipients is more likely in neighborhoods with moderate housing costs, but seems mainly dependent upon the personal contacts established between the legal advocate and the responsible nursing service (Provider D). For economically sustainable SHA concepts, the residential group needs a fraction of well-off residents that demand and pay extra services. In order to include less well-off beneficiaries, the nursing services negotiate some kind of cross-subsidization between residents (Provider D). By contrast, Provider B, located in Berlin and eastern Germany, stated that the social and economic position of people in need of care is of low relevance to nursing care providers operating SHAs in the area. Still, social assistance rates amongst SHAs remain low—except for Berlin, where rates as high as 70% can be observed (Provider B). However, many residents are likely to claim social assistance only after the housing and assistance costs of SHAs have drained their financial resources over time (i.e., not upon entry into an SHA). Provider B reported no conflicts with social assistance authorities, reflecting the different means-testing practice compared to Baden-Württemberg, but also different price structures. Provider C, meanwhile, highlighted the importance that SHAs be integrated into the neighborhood and the social environment. Provider C also offers SHAs (and assisted living) in “less wealthy” neighborhoods where housing costs are lower and the clientele are more likely to be dependent on social assistance. Hence, SHAs need not be restricted to middle-to-high socio-economic groups (Provider C).

Regarding the geographic location, SHAs (as well as other small-scale nursing concepts) were said to involve advantages for rural as well as urban areas (Provider B and C). In rural areas, providers can set up new projects combining SHAs with assisted living and day care facilities. Such projects involve efficiency gains, since care is concentrated at one location, thereby cutting down the travel time of LTC service providers in rural areas (Provider B). In urban areas, SHAs are easier to establish than nursing homes since they can use existing buildings and typically encounter fewer housing regulations. Hence, there is no obvious advantage for placing SHAs in rural areas over urban areas, and vice versa (Provider B).

Concerning cognitive and somatic problems, providers highlighted the potential of SHAs to establish a stimulating environment for people with dementia. Although SHAs are not restricted to beneficiaries with cognitive problems, Provider B highlighted that people who suffer from somatic limitations tend to value individual space more. They are less willing to accept the boundaries involved with living in residential groups, e.g., sharing a living room and bathroom with others. Dementia patients, instead, are less likely to feel restricted in SHAs and profit more from stimulation in groups:

Provider B, translated: *For example, (…) in many SHAs, residents have to share a bathroom with one or two other residents. Someone without dementia would not do that. He/She would probably not tolerate this restriction on privacy and for someone with dementia it is no problem. (…) we believe, that for the resident with dementia it (i.e., the SHA, author note) is, let’s say, a part of the therapy, if you do it right. This is not just safe keeping. And these are things which do not have the same effect on people without dementia. This has a lot to do with the cognitive changes caused by dementia and how they are stimulated by the group and how specific (negative, author note) cognitive processes are interrupted by the group and why this leads them to be in the present moment, in the here and now.*

Thus, Provider B doubted whether SHAs have the potential to create a large demand from people in need of care who are mentally competent. Rather, other forms of assisted living adjusted to individual needs will likely be the first choice amongst the larger population of LTC beneficiaries (Provider B). Providers A and C took a different position, arguing that other groups will also embrace SHA-offers in the future. Provider A especially saw potential for SHAs to serve ethnic minorities where strong family ties reflect a preference for living in larger groups, as well as can serve as a source of greater family involvement in care—a necessary ingredient for the SHA model. Provider A and D also emphasized that SHAs can be tailored according to the preferences and needs of ethnic minorities. This includes language issues, since people in old age (and even more so with cognitive problems) tend to return to their native tongue. Moreover, nursing teams which comply with cultural norms of ethnic minorities in SHAs can be employed (Provider D).

Alongside cognitive and somatic problems, providers named the severity of care needs as a problem for accessing SHAs. Since the level of dependence of LTC beneficiaries tends to increase over time, it is difficult within small-scale facilities to introduce new, less dependent people into the group. For example, it is detrimental to include people who show first signs of dementia in a group of people who suffer from severe dementia. Hence, as residential groups mature over time, with the general health status of the residents naturally declining, it becomes increasingly difficult to maintain a fitting mix of residents.

Interestingly, whereas extant literature points to the necessary role that family involvement plays in the concept of SHAs, providers did not identify family status, age, or gender as barriers to access. Concerning the former, this may be related to the fact that providers could not share information about the familial backgrounds of their residents. What providers did highlight is that where family members are present in the lives of beneficiaries, they tend to take part in decisions involving entry into SHAs, leading to longer decision-making processes. By contrast, nursing home admission is often made quickly and in response to certain catastrophic events such as accidents or a rapid decline in health.

A common theme mentioned by all providers which does not explicitly relate to equity but rather the general viability of the SHA model concerns the scarcity of qualified labor in the nursing sector. More specifically, providers argued that one advantage of the SHA model—compared to stationary care—is that qualified personnel can be used more efficiently. This helps to lessen the burden of staffing shortages experienced in nursing. At the same time, teams for assistance, nursing care, and domestic activities can be composed more easily, since there is less regulation than for residential care facilities. The latter are also strongly shaped by end-of-life-care, which entails difficulties for the staff (Provider B). Small-scale nursing projects often imply more autonomy for employees, more job satisfaction, and less stress. In particular, in rural areas, SHA concepts are perceived as a means to increase efficiency while keeping autonomy and a family-like environment for people in need of care. While all providers see some growth potential for SHA, there are diverse views on the limits. Whereas Provider A emphasizes problems of high costs and the lack of support by welfare authorities, Provider B rather perceives the concept restricted to certain groups of people in long-term care. Forms of assisted living are generally seen as the concept with the largest growth potential since economic incentives for providers meet a large demand for easy access to care tailored to individual needs. A synopsis of the interview results is provided in the following Table 2.

## 4. Discussion

The present study explored the legal provisions and expert opinions surrounding the question of equity of SHAs as an alternative model of ambulatory care within Germany. Equity, as a concept, is understood in terms of the model’s applicability and availability to a broad range of beneficiaries irrespective of socio-economic background, gender, marital status, morbidity, and/or geographic location. The results of the legal analysis point to a uniform landscape of incentives and provisions at the federal level to support the establishment of SHAs, but considerable variation emerges at the state (Länder) level of administrative law which makes for a far more complex mix of regulatory environments across the country. More specifically, in Berlin, where SHAs are most concentrated, looser regulation (concerning autonomously organized SHAs) and better financial support for SHA-residents are in place than can be seen in BW. Interestingly, while BW formally allows for provider-organized SHAs to be in operation, state administrative law creates institutional hurdles that obfuscate provider access to the market (e.g., mandating round-the-clock care and restricting providers of housing and assistance to cover nursing services as well). Moreover, resistance on the part of social assistance authorities to provide financial support to LTC beneficiaries to cover the out-of-pocket costs associated with SHA-residence create a de facto barrier to access for low socio-economic groups. In Bremen, the regulatory environment presents a kind of middle ground between Berlin and BW: whereas SHAs are legally defined in a manner similar to BW, they are subject to less monitoring and control. Moreover, in Bremen, providers are also incentivized to transform extant nursing homes into SHAs.

Taken together, findings of the legal analysis point to an especially critical role for states in determining the extent to which the SHA model is made accessible for providers and beneficiaries alike. As a result, interviewees’ perspectives on equity differ. Provider A in BW perceives considerable restrictions for those coming from lower economic backgrounds to gain access to SHAs, while Provider B in Berlin and Eastern Germany and Provider C in Bremen perceive fewer constraints. Provider D gives nuanced insights, confirming the importance of beneficiaries’ economic background, even in light of modest price structures and support from welfare authorities available in Berlin. Here, from the perspective of the nursing profession, attention is drawn to the role of personal contacts, which can facilitate the access of the less well-off to SHAs. The findings corroborate the assumption that a more restrictive regulatory environment may create barriers to access, though more systematic research is needed.

Considering other factors regarding the accessibility of the SHA model, expert interviews did not provide evidence of a significant role for family background, gender, or marital status in determining access to SHAs. This suggests that the role of familial support, often listed as an essential element of the SHA-concept, may be overrated. It also means that the secular trend of weaker family ties does not necessarily present an additional barrier to accessing care.

Concerning the expansion of SHAs to a broader population, the perspectives of interviewees vary. While all interview partners confirmed the value of the SHA concept to dementia patients, not all providers find SHAs limited to people with cognitive problems. Ethnic minorities, for example, were identified as a potential future group for which SHAs are well suited to servicing. In general, several features of SHAs and related small scale, home-like nursing concepts make them attractive to providers and beneficiaries, suggesting their further expansion in years to come. First of all, waivers from regulations applying to nursing homes favor small-scale nursing concepts. In light of workforce shortages, providers emphasize the more efficient use of nursing staff and the greater flexibility of personnel planning made possible within SHAs. Meanwhile, the current accumulation of various types of social insurance benefits contributes to the economic viability of small scale models of care. Still, it is hard to say what further expansion would mean in terms of accessibility to SHAs. Providers suggest that SHAs can be shaped in ways to include more people from lower socio-economic backgrounds. In line with our legal analysis, however, implementation remains highly dependent upon regional regulations and will require the commitment of local welfare authorities. Less can be said about take-up of the care concept by other economic and social backgrounds. Furthermore, policy feedback in response to the future expansion of SHAs will surely play a role. If SHAs turn out to be too costly for social insurance, upcoming reforms may ultimately reduce incentives for SHAs again.

## 5. Conclusions

The present study is an exploratory undertaking on which to elaborate with future research. Research is especially needed that includes more balanced analysis involving different provider perspectives, as well as the experiences of beneficiaries and family members in trying to access SHAs. While recent studies have investigated the numbers and types of SHAs emerging across Germany, relatively little attention has been paid to the demographic factors, especially economic backgrounds, characterizing their residents. In trying to understand the availability of the SHA concept to a broader population of beneficiaries, the question necessarily arises, which type of regulatory environment needs to be encouraged to make access more equitable? Provider interviews suggest that the commitment of welfare authorities to cover the costs of assistance involved with SHA residence is crucial for the inclusion of people from lower economic backgrounds. State-level regulations that govern SHAs currently play an ambivalent role. While strict regulations help to guarantee higher quality of care-concepts, they tend to increase the costs of care, thereby excluding the less well-off. Hence, quality versus access may emerge as a tradeoff. Authorities therefore need to carefully balance the standards of care and costs involved for tax payers and the insured. On the one hand, this may require a more active role of the federal government to mandate a uniform regulatory approach to SHAs across states. On the other hand, such a mandate would detract from the possibilities for policy innovation often made possible through federal experimentation. Meanwhile, given what has thus far been a rather limited application of the model to a single morbidity group—dementia sufferers—greater investment in SHAs must also be measured against other LTC needs. This said, if predictions regarding future trends in dementia prove to be accurate, this subset of the population alone will demand its own generous financing and regulation to support an alternative to care beyond the home and institutions. The findings of the present study offer a tentative yet encouraging perspective for the SHA concept to serve as one such alternative. Equitable access to the care model even for this one swelling pool of beneficiaries, however, can only be made possible if financial support is increased. Based on the variability in state level regulation across Germany at present, this would imply far greater commitment on the part of the federal government to incentivize investment and experimentation, in conjunction with local state welfare agencies, into affordable, quality care within SHAs.

## Figures and Tables

**Table 1:** German long-term care system, key objectives.

Providing social security against the risk of needing care in a manner that is akin to insurance against illness, accidents and unemployment, and protecting income in old age
Helping to reduce the physical, mental, and financial stresses related to need for care
Enabling persons to stay in their homes or to ‘age-in-place,’ as long as possible, with services based on the principles of prevention and rehabilitation before care, outpatient care before inpatient care and short stay care before full time inpatient care
Improving social security for care givers who are otherwise not employed in order to encourage people to provide care for relatives/friends; and to compensate for the effects of having to give up employment in order to become a care giver
Expanding and consolidating the care infrastructure and encouraging competition amongst service providers [8]

**Table 2:** Summary of interview results.

Findings Related to:	Main Findings
● Economic background	Low income is a crucial factor reducing the chances of access. However, the role of income and need of social assistance is dependent upon local practices of welfare authorities, local cost structures and personal contacts.
● Social position	The role of social class and educational background has been most important in the early days of the shared housing arrangement (SHA) concept. It has lost importance with the evolution SHAs that are increasingly initialized or organized by professional providers of care.
● Family background	Family support and strong family ties help to establish and run SHAs, however family support often remains passive and it is difficult to claim active participation by SHA residents and providers.
● Level and form of dependency	The SHA concept is particularly well suited for people suffering from dementia. SHAs are said to provide positive stimulation for this group of dependents. Yet, providers hold different opinions concerning the question as to whether SHAs are restricted to people with dementia.
● Age and sex	No implications revealed.
● Ethnic background	Providers consider SHAs as a concept of care which can be well adapted to the strong family ties of ethnic groups and provides opportunities to adjust care giving to the specific needs and preferences of ethnic minorities.
● Geographic location	SHAs can be successfully established in rural as well as urban areas. The regional differences in the concentration of SHAs are to some extent related to differences in regulation and means-testing practices of local welfare authorities. The latter needs further research.
